# Self-Transforming Configuration Based on Atmospheric-Adaptive Materials for Solid Oxide Cells

**DOI:** 10.1038/s41598-018-35659-y

**Published:** 2018-11-21

**Authors:** Seona Kim, Seungtae Lee, Junyoung Kim, Jeeyoung Shin, Guntae Kim

**Affiliations:** 1 Department of Energy Engineering, Ulsan National Institute of Science and Technology (UNIST) Ulsan, Ulsan, 44919 Republic of Korea; 2 Division of Mechanical Systems Engineering, Sookmyung Women’s University Seoul, Seoul, 04310 Republic of Korea; 3 Institute of Advanced Materials and Systems, Sookmyung Women’s University Seoul, Seoul, 04310 Republic of Korea

## Abstract

Solid oxide cells (SOC) with a symmetrical configuration have been focused due to the practical benefits of such configurations, such as minimized compatibility issues, a simple fabrication process and reduced cost compared to SOCs with the asymmetrical configuration. However, the performance of SOCs using a single type of electrode material (symmetrical configuration) is lower than the performance of those using the dissimilar electrode materials (asymmetrical configuration). Therefore, to achieve a high-performance cell, we design a ‘self-transforming cell’ with the asymmetric configuration using only materials of the single type, one based on atmospheric adaptive materials. Atmospheric-adaptive perovskite Pr_0.5_Ba_0.5_Mn_0.85_Co_0.15_O_3-*δ*_ (PBMCo) was used for the so-called self-transforming cell electrodes, which changed to layered perovskite and metal in the fuel atmosphere and retained its original structure in the air atmosphere. In fuel cell mods, the self-transforming cell shows excellent electrochemical performance of 1.10 W cm^−2^ at 800 °C and good stability for 100 h without any catalyst. In electrolysis mode, the moderate current densities of −0.42 A cm^−2^ for 3 vol.% H_2_O and −0.62 A cm^−2^ for 10 vol.% H_2_O, respectively, were observed at a cell voltage of 1.3 V at 800 °C. In the reversible cycling test, the transforming cell maintains the constant voltages for 30 h at +/− 0.2 A cm^−2^ under 10 vol. % H_2_O.

## Introduction

Among the renewable energy conversion systems, hydrogen energy systems have received tremendous attention because hydrogen is a versatile, clean, efficient fuel, and the most abundant element in the universe^1^. Solid oxide fuel cells (SOFCs) are one of the hydrogen energy systems offering exceptionally high conversion efficiency, low pollution emission and excellent fuel flexibility^2–4^. Furthermore, SOFCs can be used as solid oxide electrolysis cells (SOECs) by adding H_2_O under reverse operation, represented as solid oxide cells (SOCs)^5–8^.

Conventional SOCs consist of a dense electrolyte and dissimilar materials for the anode and cathode, creating the so-called ‘asymmetrical configuration’. Asymmetrical configurations are advantageous for performance because they use designated electrodes for cathode and anode, respectively. The use of dissimilar electrode materials, however, gives rise to problems such as complex fabrication processes and corresponding expensive fabrication cost, and poor thermo-mechanical compatibility due to the different thermal expansion coefficients^3,9,10^. Furthermore, asymmetrical configurations require additional materials for the buffer layer in order to reduce thermal problems. Asymmetrical configurations have thus been difficult to commercialize despite their remarkable electrochemical performance.

Another configuration called ‘symmetrical configuration’ has been suggested by applying the identical materials to both electrodes (*i.e*. cathode and anode). By employing identical interfaces between electrode-electrolyte, the symmetrical configuration diminishes the compatibility problem of the asymmetrical configuration. Also, the symmetrical configuration is advantageous for practical applications due to its simple fabrication process and cost reduction obtained by conducting only one thermal step for the electrodes fabrication. Even with these benefits, the cell with symmetrical configuration has yet to achieve electrochemical performance comparable with asymmetrical configuration because it is difficult to find materials having the necessary structural and chemical stability and high catalytic activity in both oxidizing and reducing conditions^11–15^.

**Figure 1:**
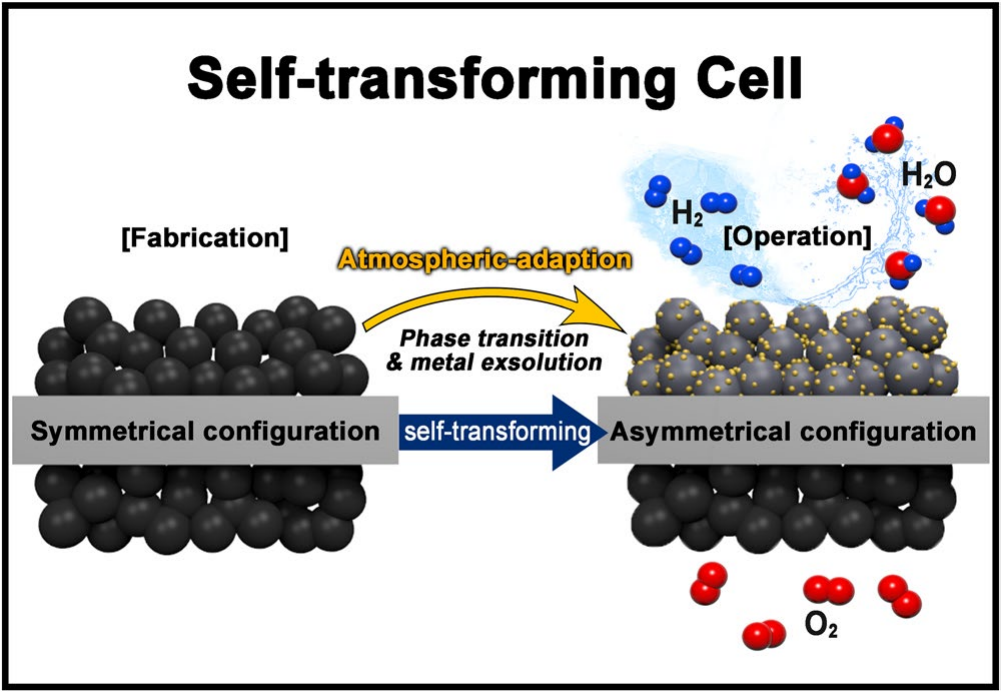
Schematic illustration presenting the concept of transforming cell.

Like this, the SOC has a configuration dilemma; specifically, there is a trade-off relationship between the optimizing catalytic activity and the practical applicability. Thus, we introduce the ‘transforming cell’ based on the atmospheric-adaptive materials to take full advantage of both asymmetrical and symmetrical configuration. The atmospheric-adaptive materials refer to the material which changes the structural properties (e.g. phase and composition) by themselves to have the high catalytic activity depending on the atmosphere. Thus, the transforming cell can be fabricated with symmetrical configuration and can be operated with the asymmetric configuration, as illustrated in Fig. 1, providing excellent electrochemical performance and few thermal problems with a simple fabrication process.

As a candidate for the electrode material, Pr_0.5_Ba_0.5_MnO_3-*δ*_ (PBMO) was considered due to its excellent chemical and structural stability and the mixed valence Mn^4+^/Mn^3+^/Mn^2+^ which can accept electrons to promote the reaction^10,16^. Also, the catalytic activities can be tailored by A- and B- site doping because the perovskite oxide can choose its elements flexibly. Recently, Kwon *et al*. reported the exsolution phenomenon in PBMO systems, with the substitution of transition metal for Mn. Based on previous studies, among the transition metals, cobalt is the most versatile as a dopant to both air electrodes and fuel electrodes with doping and exsolution. Generally, cobalt doping on air electrode material improves the electrical conductivity by increasing the oxygen vacancy concentration^17,18^. For the fuel electrode, the exsolved cobalt plays the role of a catalyst, enhancing the electrochemical performance^19–21^. In summary, Pr_0.5_Ba_0.5_Mn_0.85_Co_0.15_O_3-*δ*_ (PBMCo) has simple perovskite structure with mixed cubic and hexagonal layers^19,22–26^. Exposed to the hydrogen, the structure is changed to the cation-ordered perovskite with Co exsolution.

Thus, Pr_0.5_Ba_0.5_Mn_0.85_Co_0.15_O_3-*δ*_ (PBMCo) was chosen as the atmospheric-adaptive material to obtain the cobalt doping effect on air electrodes and the cobalt exsolution effect on fuel electrodes. To increase the surface area and corresponding electrochemical performance of the electrodes, the infiltration technique was applied as a fabrication method. As expected, with the derived electrode materials, the transforming cell shows competitive electrochemical performance and promising stability compared to other symmetrical cells reported in the literature.

## Results and Discussions

**Figure 2:**
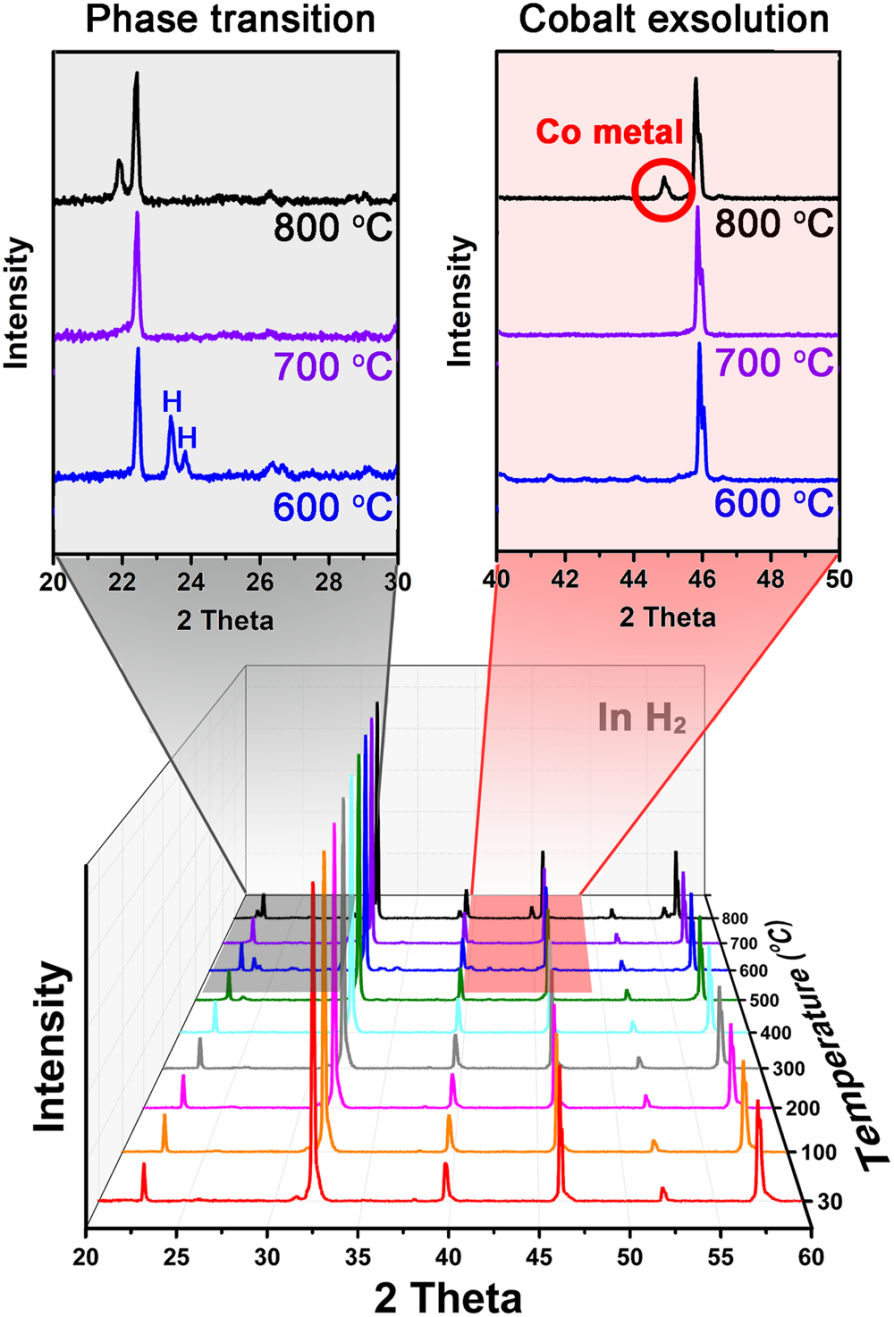
X-ray diffraction (XRD) patterns obtained through *in-situ* annealing of a pre-sintered PBMCo in the range 20° < 2θ < 60°. The sample was held at each temperature for 2 h in hydrogen.

The operating conditions of the transforming cell were determined by conducting *in-situ* X-ray diffraction (XRD) under hydrogen with the pre-sintered Pr_0.5_Ba_0.5_Mn_0.85_Co_0.15_O_3-*δ*_ (PBMCo)-LSGM electrode. As shown in Fig. 2, PBMCo has simple perovskite mixed cubic and hexagonal layers at temperatures under 600 °C. A layered perovskite structure was successfully with the disappearance of the hexagonal peaks in the temperature range from 700 °C to 800 °C. The metal peak was observed at around 2θ = 45° at 800 °C, indicating the exsolved cobalt. Taking these observations into consideration, the fuel electrode was reduced at 800 °C for subsequent measurements.

**Figure 3:**
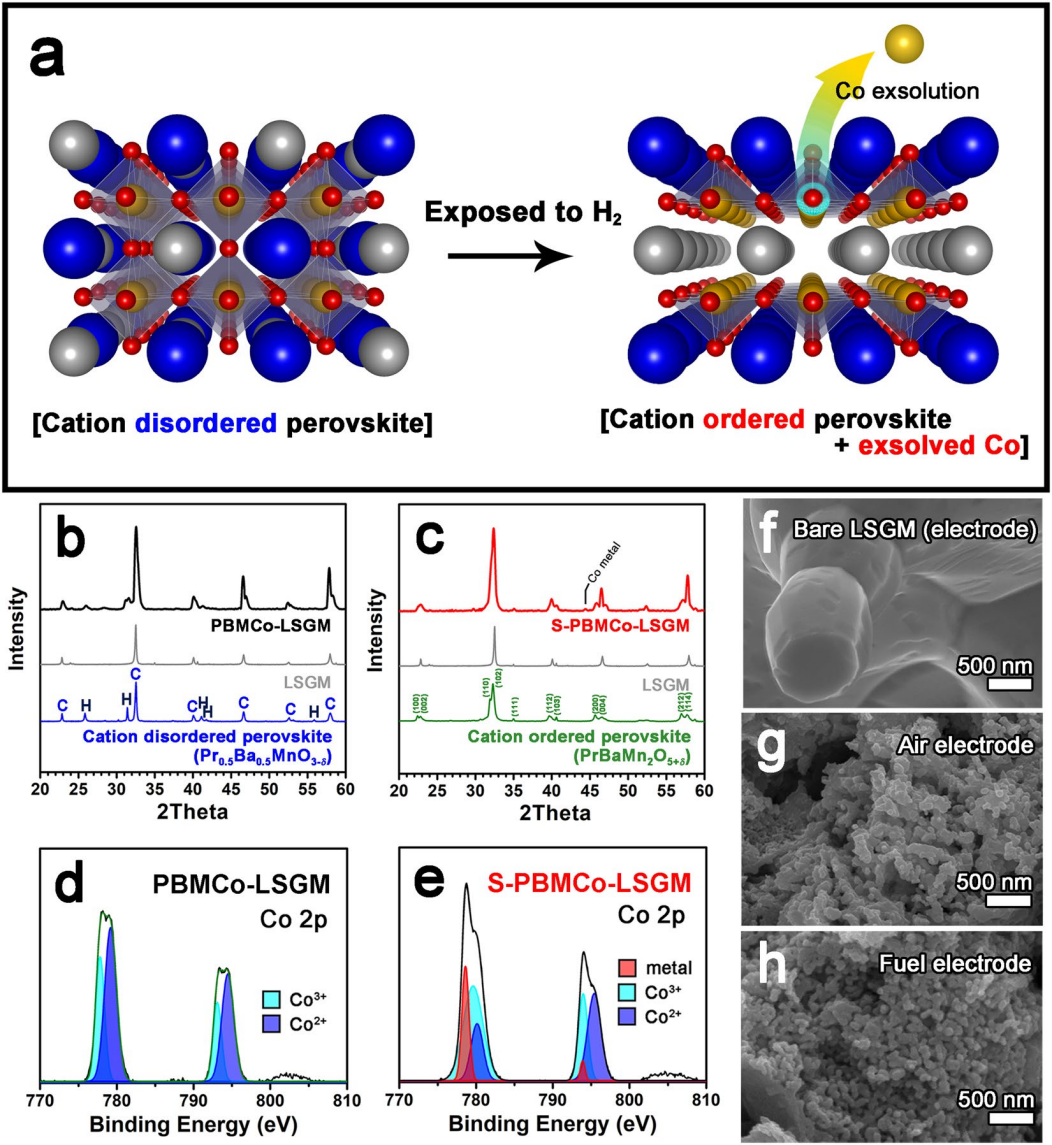
(**a**) Schematic illustrations of the transforming cell in fabrication and in operation with phase transition of PBMCo. XRD patterns of (**b**) PBMCo-LSGM (sintered in air) and (**c**) S-PBMCo-LSGM (reduced in hydrogen) in range of 20° < 2θ  < 60^o^. Co 2p electron region of X-ray photoelectron spectroscopy (XPS) profiles of (**d**) PBMCo-LSGM and (**e**) S-PBMCo-LSGM. Scanning electron microscopy (SEM) images of (**f**) bare LSGM, (**g**) PBMCo-LSGM, and (**h**) S-PBMCo-LSGM electrodes.

Figure 3a illustrates the phase transition and metal exsolution of atmospheric-adapted material PBMCo. During fabrication, the air and fuel electrodes sintered in air show simple perovskite structure, implying cobalt-doped PBMO (Pr_0.5_Ba_0.5_Mn_0.85_Co_0.15_O_3-*δ*_, PBMCo). In operation, the fuel electrode was exposed to an H_2_ atmosphere and thus, specialized by phase transition and cobalt metal exsolution (PrBaMn_1.7_Co_0.3-*x*_O_5+*δ*_ + *x*Co, S-PBMCo), while the air electrode retained the simple perovskite structure. To confirm the phase transition and the cobalt exsolution, X-ray diffraction (XRD) and X-ray photoelectron spectroscopy (XPS) were conducted. In Fig. 3b,c, XRD patterns identify the phases of the as-sintered samples before and after reduction in H_2_ at 800 °C, representing PBMCo-LSGM and S-PBMCo-LSGM, respectively. Before the reduction, the XRD patterns can be well-indexed to the crystal planes of simple perovskite and LSGM without any undesired reaction. After reduction, the patterns involve the phases of cation-ordered perovskite and LSGM, combined with peaks of cobalt metal caused by the exsolution phenomenon. These results are in good agreement with the expectation that the cobalt is doped in PBMO at the air-electrode side and is exsolved at the fuel-electrode side during operation.

In Figure 3d,e, the surface electronic states of cobalt were analyzed with XPS profiles of Co2p for PBMCo-LSGM and S-PBMCo-LSGM. The binding energy (BE) was calibrated with the C1s peak at 284.5 eV. Considering the overlap between the peaks of the Ba 3d and Co 2p, the Ba 3d peaks of Pr_0.5_Ba_0.5_MnO_3_ and PrBaMn_2_O_5_ were analyzed to observe the change of Ba 3d during reduction and the corresponding phase transition. The XPS results for Pr_0.5_Ba_0.5_MnO_3_ and PrBaMn_2_O_5_ are presented in Fig. S1, implying that the Ba 3d peaks are independent of the phase transition. In this regard, changes in the XPS peak at a BE range of 770–810 eV demonstrate a change in the Co 2p peak during the phase transition of PBMCo and S-PBMCo. Based on the fitted results, the Co^3+^/Co^2+^ increases from 0.56 for PBMCo to 1.43 for S-PBMCo can be attributed to lattice defects and corresponding cobalt oxidation (Co^2+^ → Co^3+^). Additionally, the cobalt oxidation was also supported by the magnesium reduction (Mn^3+/4+^ → Mn^2+^) as confirmed by the XPS results of the Mn 2p_3/2_ peaks for PBMCo and S-PBMCo in Fig. S2. The appearance of cobalt metal peaks in S-PBMCo demonstrates that the cobalt exsolution occurs under an operating condition (H_2_, 800 °C). A cross-sectional view of the tri-layer cell (PBMCo-LSGM/LSGM/S-PBMCo-LSGM) is shown in Fig. S3. The dense LSGM electrolyte was about 60 μm thick and appeared to be in good connection with the electrode layer. The scanning electron microscopy (SEM) images of the electrodes (Fig. 3f–h) show that the nano-sized particles are formed and uniformly distributed on the LSGM, retaining its morphology even after reduction. These microstructural properties of the cell with the nanostructured electrodes and the thin electrolyte layer are expected that the self-transforming cells will show high electrochemical performance.

For the electrochemical analysis, the impedance spectra (Fig. S4) of the transforming cells were measured at 700, 750 and 800 °C. The ohmic loss of the cell was measured by the high-frequency intercept with the real axis to be 0.147, 0.187, and 0.244 Ω cm^2^ at 800, 750, 700 °C, respectively. The polarization resistance originated from the electrodes was determined by the difference between the two intercepts on the real axis to be 0.139, 0.273, and 0.530 Ω cm^2^ at 800, 750, 700 °C, respectively. To estimate the cathodic loss, we conducted the half-cell test using the PBMCo-LSGM/LSGM/PBMCo-LSGM cell. Figure S5a shows the Nyquist plots of PBMCo-LSGM at operating temperatures of 700, 750, and 800 °C, respectively, with the fitted line by the software EC-Lab. The ohmic resistance was eliminated to direct comparison of cathodic polarization resistance at the various operating temperature. The cathodic R_p_ values of the PBMCo-LSGM were 0.033, 0.073, and 0.189 Ω cm^2^ at 800, 750, and 700 °C, respectively. The fitted lines were in good agreement with the Nyquist plots on the basis of the equivalent circuit in the inset of Fig. S5b. In the equivalent circuit, L is an inductance caused by the cables; R_1_ is ohmic resistance; R_2_ is associated with the charge transfer during the migration and diffusion of oxygen ion from the reaction site into electrolyte lattice; R_3_ is induced by the non-charge transfer related to oxygen surface exchange, solid-state diffusion, and gas-phase diffusion. The specific values of R_2_, R_3_ and R_p_ (R_2_ + R_3_) are also summarized in Table S1.

**Figure 4:**
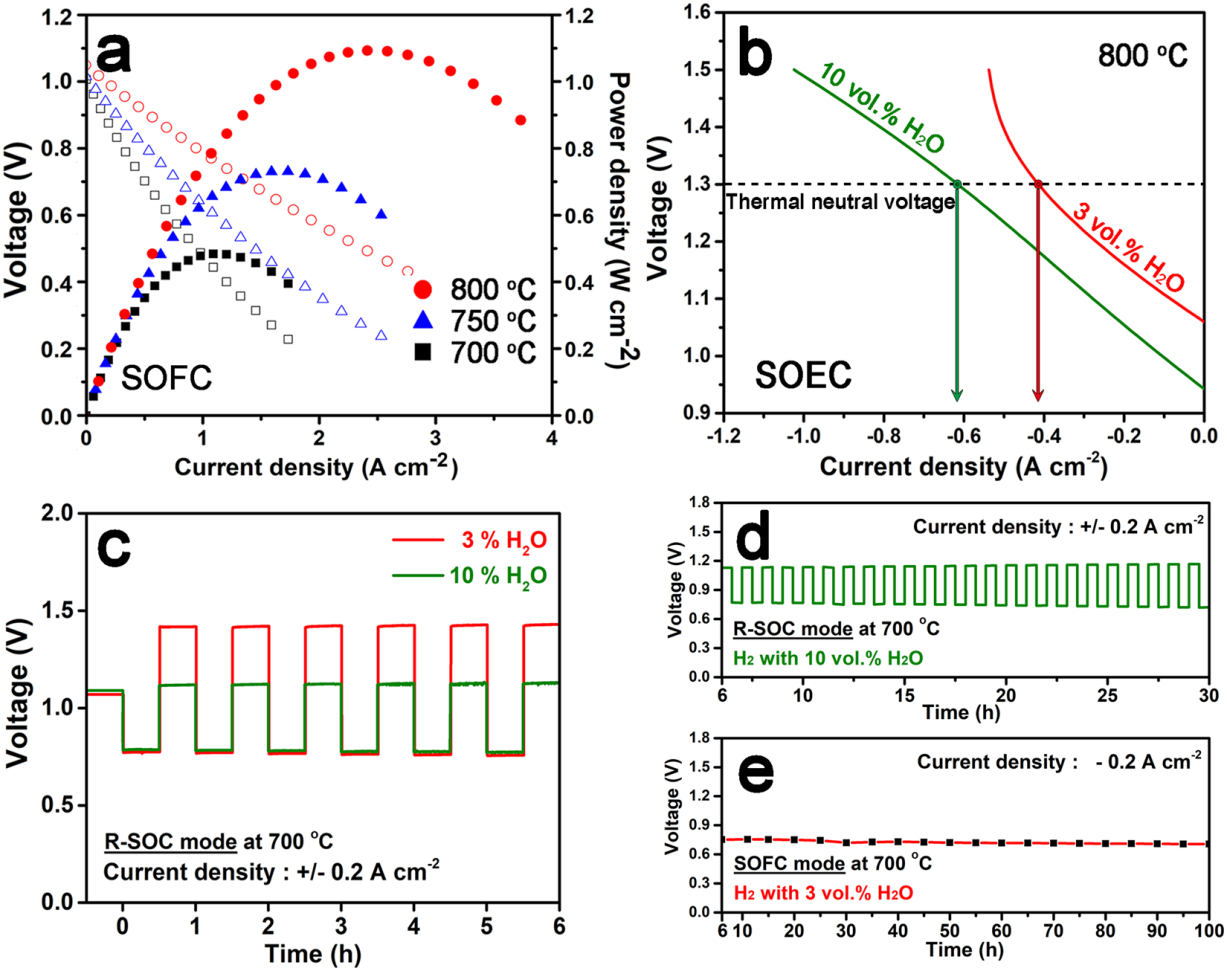
(**a**) Voltage-current density and corresponding power density curves of the transforming cell at 800, 750, 700 °C using humidified H_2_ (3% H_2_O). (**b**) Voltage-current density curves of the transforming cell at 800 ^o^C under electrolysis mode with 3% and 10% H_2_O containing H_2_ safe gas. (**c**) Long-term stability of the transforming cell at 700 °C with current density of −0.2 A cm^−2^. (**d**) The reversible cycling result performed at −0.2 A cm^−2^ (electrolysis mode) and at 0.2 A cm^−2^ (fuel cell mode).

Figure 4a shows the power density and voltage as functions of current density for the transforming cell with humidified H_2_ as fuel and air as the oxidant. As expected from low polarization resistance, the transforming cell presents remarkably high maximum power density of 1.10 W cm^−2^ at 800 °C without any additional catalyst in comparison with the experimental values of the symmetrical cell (Table S3)^3,10–12,14,16,27–31^. Consequently, the use of the atmospheric-adaptive electrode (PBMCo) provides excellent catalytic activity for each atmosphere by tailoring the structure itself. Such excellent performance should be attributed from the novel structure of infiltration and the thin electrolyte. Moreover, the transforming cell shows potential as an electrolysis cell, as shown in Fig. 4b. The electrochemical performance was recorded by scanning current density at the voltage range from 0 to 1.5 V with 3% H_2_O and 10% H_2_O (H_2_ safe gas included), respectively. Even the small amount of H_2_O was applied, the transforming cell shows the reasonable electrochemical performance of −0.42 A cm^−2^ for 3 vol.% H_2_O and −0.62 A cm^−2^ for 10 vol.% H_2_O, respectively, at a cell voltage of 1.3 V at 800 °C. To evaluate the reversibility of operation as SOCs, the cycling between electrolysis mode at −0.2 A cm^−2^ and fuel cell mode at +0.2 A cm^−2^ was performed at 700 °C for 3 and 10 vol. % H_2_O, respectively. At both H_2_O amount, the cell shows a stable performance in electrolysis and fuel cell modes without detectable degradation (Fig. 4c). The cell under 10 vol. % H_2_O maintained the constant voltages in both electrolysis and fuel cell modes for 30 h (Fig. 4d). After reversible cycling for 6 h, the cell under 3 vol. % H_2_O was operated under SOFC modes with 3 vol. % H_2_O without any critical degradation during ~100 h (Fig. 4e).

## Conclusion

To take full advantage of both asymmetrical and symmetrical configurations, the transforming cell was designed by applying the atmospheric-adaptive material Pr_0.5_Ba_0.5_Mn_0.85_Co_0.15_O_3-*δ*_ (PBMCo) to the electrodes. The PBMCo at anode was specialized into PrBaMn_1.7_Co_0.3-*x*_O_5+*δ*_ + *x*Co (S-PBMCo) by phase transition and metal exsolution as confirmed by XRD and XPS. The transforming cell was operated in the SOFC and SOEC modes. In SOFC modes, the transforming cell shows high maximum power density of 1.10 W cm^−2^ at 800 °C without any additional catalyst and highly stable performance during 100 h. In SOEC mode, the transforming cell shows reasonable electrochemical performance of −0.62 A cm^−2^ for 10 vol.% at a cell voltage of 1.3 V at 800 °C. For reversible cycling test, the transforming cell maintains the constant voltages for 30 h at +/− 0.2 A cm^−2^ under 10 vol. % H_2_O. These results indicate that the transforming cell is a promising system for solid oxide cells as both fuel cells and electrolysis cells.

## Methods

### Preparation of the transforming cell

For the infiltration process, the preparation is just scaffold and precursor solution. The triple-layer cell (porous | dense | porous) was prepared via dual-tape casting as scaffold composed of ionic conductor. First, La_0.9_Sr_0.1_Ga_0.8_Mg_0.2_O_3-*δ*_ (LSGM) powder was prepared by solid-state reaction. For the slurry of dual-tape casting, the LSGM powder was added into ethanol with brown menhaden fish oil (Tape casting warehouse), xylene (Aldrich, 98.5 + %), polyalkylene glycol (Tape casting warehouse), butyl benzyl phthalate (Tape casting warehouse), and polyvinyl butyral (Tape casting warehouse). A green tape containing 50 wt.% LSGM and 50 wt.% graphite (for the porous layer) was cast on the top of the 100 wt.% LSGM tape. Sequentially, the punched tape for porous layer was laminated with the punched co-cast tape. Then triple-layer tapes were fired at 1475 °C for 5 h. The precursor solution was prepared by dissolving the quantitative amount of Pr(NO_3_)_3_·6H_2_O (Aldrich, 99.9%), Ba(NO_3_)_2_ (Aldrich, 99 + %), Mn(NO_3_)_2_ (Aldrich, 98%), Co(NO_3_)_2_·6H_2_O (Aldrich, 98 + %) and citric acid into distilled water. To make a high concentration solution, the ammonia is added to the precursor solution after heat treatment reducing the amount of solvent. The high concentration solution was infiltrated into the porous side of the triple-layer cell and calcined at 450 °C for 20 min. This was repeated up to 45 wt.% loading amounts.

### Measurements

For the structural identification, X-ray diffraction (XRD) (Bruker D8 Advance) were conducted in a 2θ range from 20° to 60°. The microstructure was observed using scanning electron microscopy (SEM) (Nova SEM) and transmission electron microscopy (TEM) (JEOL, JEM-2100F). X-ray photoelectron spectroscopy (XPS) spectra were taken using ESCALAB 2 50XI from Thermo Fisher Scientific with a monochromated Al-Kα (ultraviolet He1, He2) X-ray source. To measure the electrochemical performance, the standard four-probe technique was employed with a BioLogic Potentiostat. The sample was prepared by attaching silver wires to both electrodes of the electrolyte supported cell as current collectors with silver paste. Then the cell was sealed onto a 10 mm diameter alumina tube with ceramic adhesive (Aremco, Ceramabond 552). For the electrochemical performance test on the fuel cell mode, the humidified hydrogen (3 vol.% H_2_O) and air were fed to the fuel electrode side and the air electrode side, respectively. On the electrolysis cell mode, the hydrogen containing 10 vol. % H_2_O was applied to the fuel electrode side. At the same time, area specific resistance (ASR) was measured by AC impedance spectroscopy at open circuit voltage (OCV) in the 10^−1^−10^4^ Hz frequency range and was calculated with an active electrode area of 0.36 cm^2^.

## Electronic supplementary material

Supporting information

## Data Availability

The data that support the findings of this study are available from the corresponding authors upon reasonable request.

## References

[1] Obama B (2017). The irreversible momentum of clean energy. science.

[2] Kim J-H, Manthiram A (2015). Layered LnBaCo2O5 + δ Perovskite Cathodes for Solid Oxide Fuel Cells: An Overview and Perspective. J Mater Chem A.

[3] Kim S (2015). Nanostructured Double Perovskite Cathode With Low Sintering Temperature For Intermediate Temperature Solid Oxide Fuel Cells. ChemSusChem.

[4] Stambouli A, Traversa E (2002). Solid oxide fuel cells (SOFCs): A review of an environmentally clean and efficient source of energy. Renewable and Sustainable Energy Reviews.

[5] Minh NQ, Mogensen MB (2013). Reversible Solid Oxide Fuel Cell Technology for Green Fuel and Power Production. The electrochemical Society Interface.

[6] Jensen SH (2015). Large-scale electricity storage utilizing reversible solid oxide cells combined with underground storage of CO 2 and CH 4. Energy Environ Sci.

[7] Jun A, Kim J, Shin J, Kim G (2016). Achieving High Efficiency and Eliminating Degradation in Solid Oxide Electrochemical Cells Using High Oxygen-Capacity Perovskite. Angewandte Chemie - International Edition.

[8] Kim J (2017). Hybrid-solid oxide electrolysis cell: A new strategy for efficient hydrogen production. Nano Energy.

[9] Shao Z, Haile SM (2004). A high-performance cathode for the next generation of solid-oxide fuel cells. Nature.

[10] Kim S (2017). Tailoring Ni-based catalyst by alloying with transition metals (M = Ni, Co, Cu, and Fe) for direct hydrocarbon utilization of energy conversion devices. Electrochimica Acta.

[11] Liu Q, Dong X, Xiao G, Zhao F, Chen F (2010). A novel electrode material for symmetrical SOFCs. Advanced Materials.

[12] Ruiz-Morales JC (2007). LSCM-(YSZ-CGO) composites as improved symmetrical electrodes for solid oxide fuel cells. Journal of the European Ceramic Society.

[13] Buyukaksoy A, Petrovsky V, Dogan F (2013). Solid Oxide Fuel Cells with Symmetrical Pt-YSZ Electrodes Prepared by YSZ Infiltration. Journal of the Electrochemical Society.

[14] Choi S (2016). A robust symmetrical electrode with layered perovskite structure for direct hydrocarbon solid oxide fuel cells: PrBa 0.8 Ca 0.2 Mn 2 O 5 + δ. J Mater Chem A.

[15] Liu Q, Yang C, Dong X, Chen F (2010). Perovskite Sr2Fe1.5Mo0.5O6-δas electrode materials for symmetrical solid oxide electrolysis cells. International Journal of Hydrogen Energy.

[16] Information S (2014). Layered oxygen-deficient double perovskite as an efficient and stable anode for direct hydrocarbon solid oxide fuel cells. Nature Materials.

[17] Pan X (2013). Effect of Co doping on the electrochemical properties of Sr2Fe1.5Mo0.5O6 electrode for solid oxide fuel cell. International Journal of Hydrogen Energy.

[18] Gu H (2008). Effect of Co doping on the properties of Sr0.8Ce0.2MnO3-δ cathode for intermediate-temperature solid-oxide fuel cells. International Journal of Hydrogen Energy.

[19] Kwon O (2017). Exsolution trends and co-segregation aspects of self-grown catalyst nanoparticles in perovskites. Nature Communications.

[20] Cui S-H (2013). Cobalt doped LaSrTiO3−δ as an anode catalyst: effect of Co nanoparticle precipitation on SOFCs operating on H2S-containing hydrogen. Journal of Materials Chemistry A.

[21] Sun Y-FF (2016). New Opportunity for *in Situ* Exsolution of Metallic Nanoparticles on Perovskite Parent. Nano Letters.

[22] Iorgulescu M (2013). Mixed metallic Ba(Co,Mn)X0.2-xO3-δ(X = F, Cl) hexagonal perovskites. Journal of Solid State Chemistry.

[23] Miranda L (2007). Structural chemistry and magnetic properties of the BaMn 0.4Co0.6O2.83 hexagonal perovskite. Chemistry of Materials.

[24] Miranda L (2008). Study of the structural, magnetic, and electrical properties of the 5H hexagonal-type perovskite BaMn0.2Co0.8O2.80. Chemistry of Materials.

[25] Modeshia DR, Walton RI (2010). Solvothermal synthesis of perovskites and pyrochlores: Crystallisation of functional oxides under mild conditions. Chemical Society Reviews.

[26] Zhang Y-Q, Sun Y-F, Luo J-L (2016). Developing Cobalt Doped Pr0.5Ba0.5MnO3-δ Electrospun Nanofiber Bifunctional Catalyst for Oxygen Reduction Reaction and Oxygen Evolution Reaction. ECS Transactions.

[27] Lin B, Wang S, Liu X, Meng G (2010). Simple solid oxide fuel cells. Journal of Alloys and Compounds.

[28] Zhang Y, Zhou Q, He T (2011). La0.7Ca0.3CrO3-Ce0.8Gd 0.2O1.9 composites as symmetrical electrodes for solid-oxide fuel cells. Journal of Power Sources.

[29] Ruiz-Morales JC, Canales-Vázquez J, Peña-Martínez J, López DM, Núñez P (2006). On the simultaneous use of La0.75Sr0.25Cr0.5Mn0.5O3-?? as both anode and cathode material with improved microstructure in solid oxide fuel cells. Electrochimica Acta.

[30] El-Himri A, Marrero-López D, Ruiz-Morales JC, Peña-Martínez J, Núñez P (2009). Structural and electrochemical characterisation of Pr0.7Ca0.3Cr1−yMnyO3−δ as symmetrical solid oxide fuel cell electrodes. Journal of Power Sources.

[31] Scribe P (2006). Double Perovskites as Anode. Science.

