# Supplementary material for: Self-Transforming Configuration Based on Atmospheric-Adaptive Materials for Solid Oxide Cells
Source: Sci Rep. 2018 Nov 21;8:17149. doi: 10.1038/s41598-018-35659-y (PMC6249295; doi:10.1038/s41598-018-35659-y)
Supplement: Supplementary file 1 — Supporting information [file 41598_2018_35659_MOESM1_ESM.docx]

Supporting Information

**Self-Transforming Configuration Based on Atmospheric-Adaptive Materials for Solid Oxide Cells**

Seona Kim, Seungtae Lee, Junyoung Kim, Jeeyoung Shin*, and Guntae Kim*

.


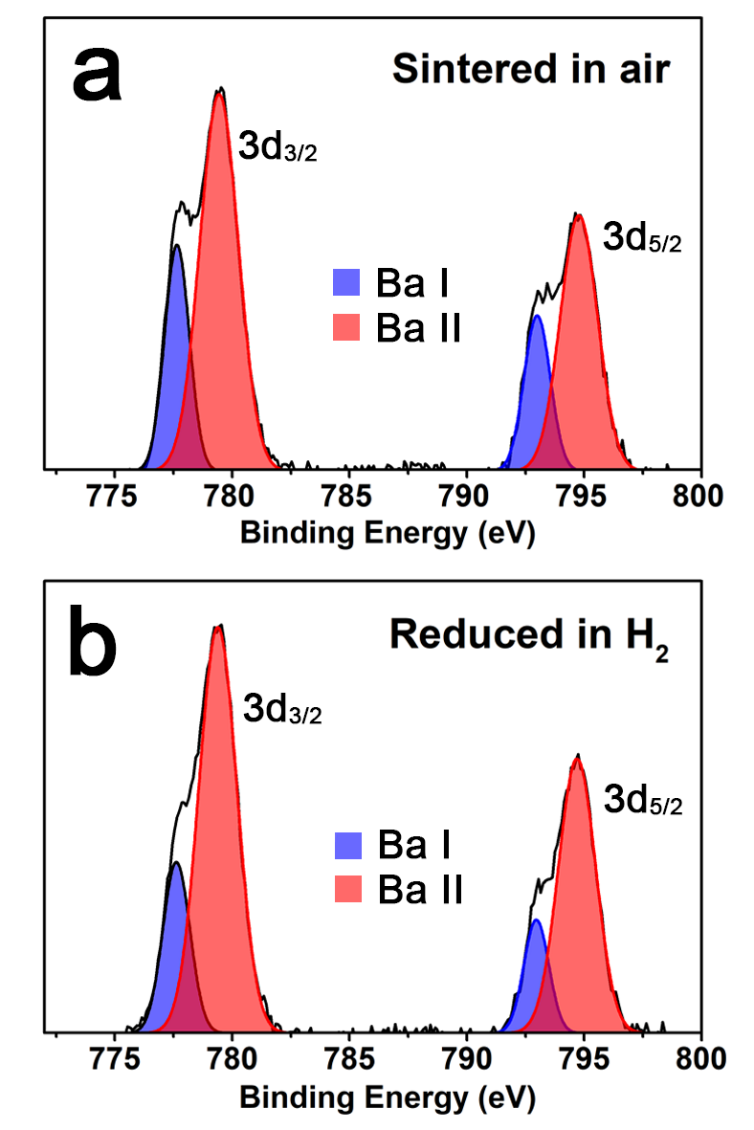


**Figure S1.** XPS spectra of the Ba 3d peaks for (a) Pr_0.5_Ba_0.5_MnO_3_(before reduction) (b) PrBaMn_2_O_5_ (after reduction).


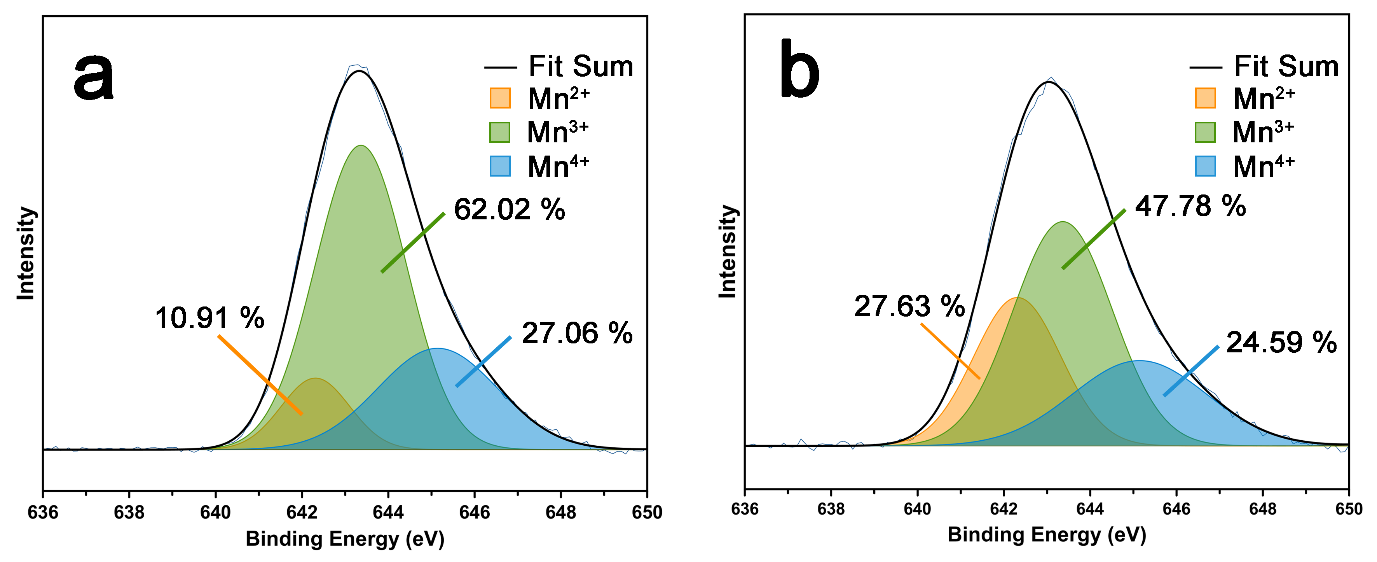


**Figure S2.** XPS spectra of the Mn 2p_3/2_ peaks for (a) Pr_0.5_Ba_0.5_Mn_0.85_Co_0.15_O_3_(before reduction) (b) PrBaMn_1.7_Ba_0.3_O_5_ (after reduction).


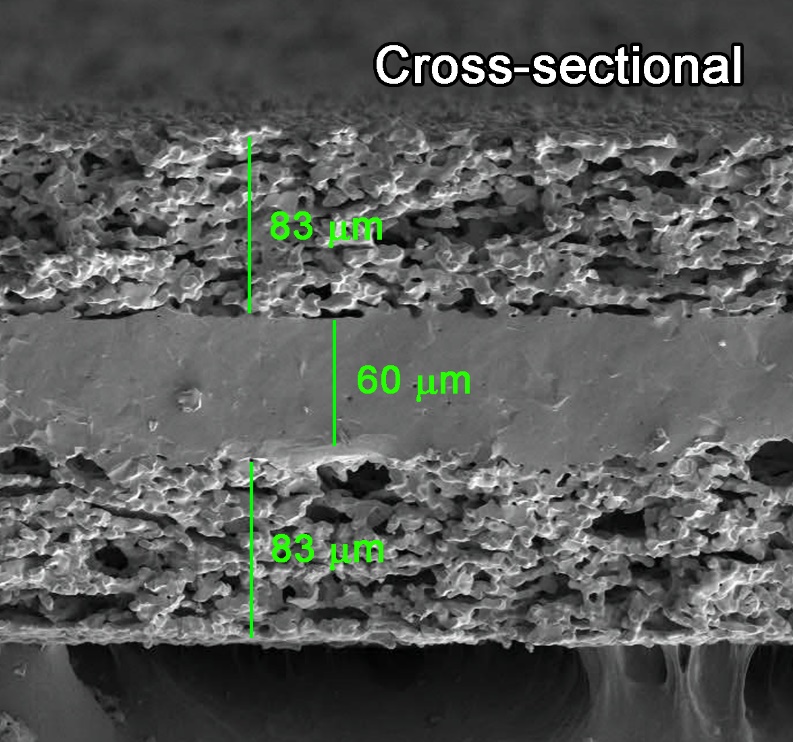


**Figure S3.** Cross sectional scanning electron microscopy (SEM) image of trple-layer LSGM electrolyte-supported cell with the configuration of porous LSGM | dense LSGM | porous LSGM).


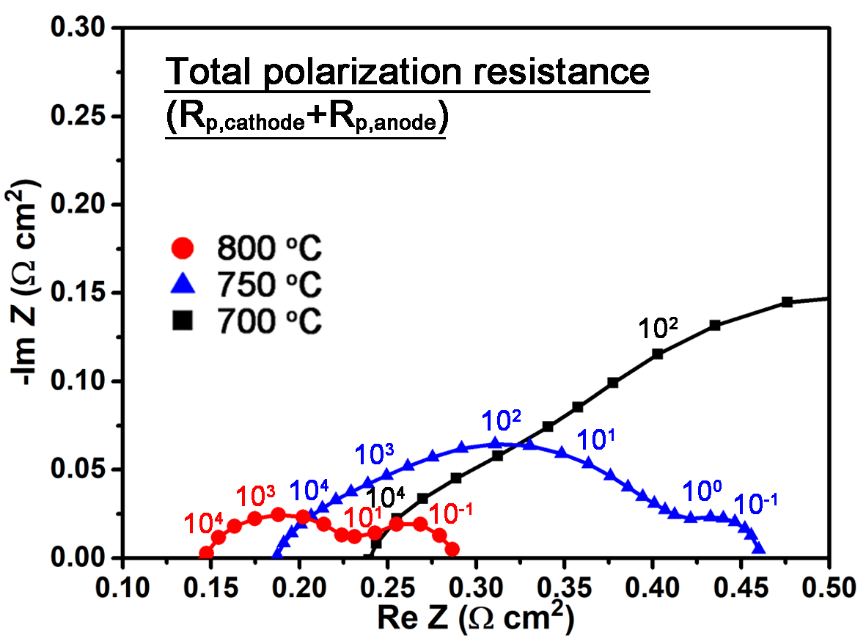


**Figure S4**. Impedance spectra of the transforming cell with the configuration of PBMCo-LSGM/LSGM/S-PBMCo-LSGM using H_2_ as fuel and air as the oxidant.


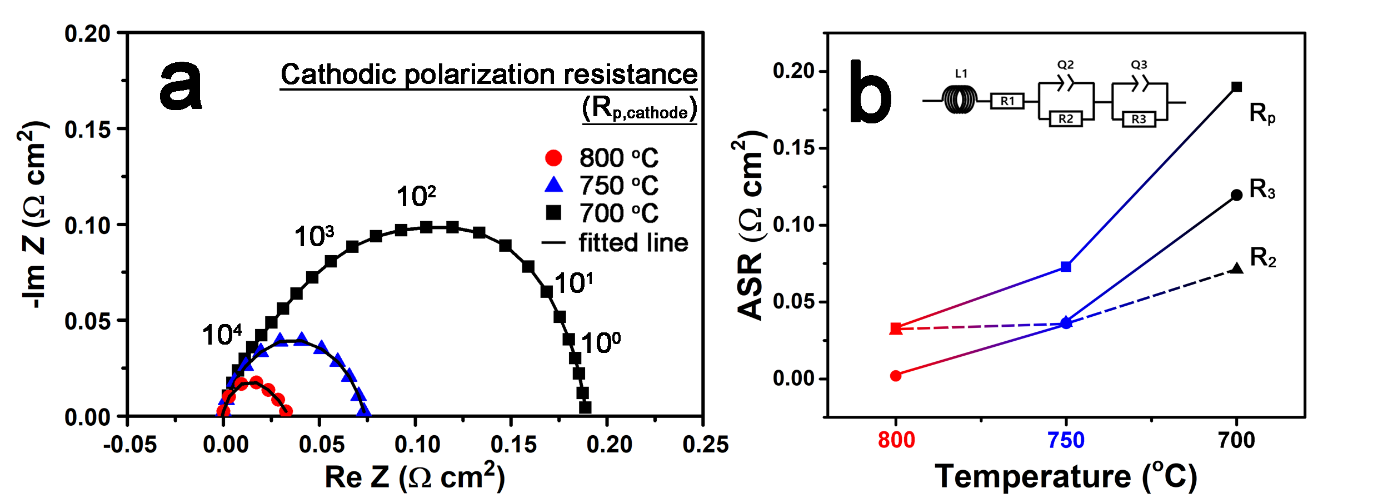


**Figure S5.** (a) Experimental and simulated Nyquist plots of PBMCo-LSGM composite on a LSGM symmetric cell. (b) Comparison of R_2_, R_3_, and R_p_ for PBMCo-LSGM composites on a LSGM symmetric cell based on the fitting result by the equivalent circuit shown as an inset

**Table S1.** Electrochemical impedance spectroscopy fitting results of PBMCo-LSGM measured at various temperature in air

| Temp. [^o^C] | Half-cell test [Ω cm^2^] | | | |
| --- | --- | --- | --- | --- |
|  | Measured | Fitted | | |
|  | Cathodic loss (R_p_) | R_2_ | R_3_ | R_p_ |
| 800 | 0.033 | 0.031 | 0.002 | 0.033 |
| 750 | 0.073 | 0.036 | 0.036 | 0.072 |
| 700 | 0.189 | 0.071 | 0.118 | 0.189 |

**Table S2.** Electrochemical impedance spectroscopy results

| Temp. [^o^C] | Transforming cell test | | Half-cell test |
| --- | --- | --- | --- |
|  | Ohmic resistance  [Ω cm^2^] | Polarization resistance  [Ω cm^2^] | Cathodic loss  [Ω cm^2^] |
| 800 | 0.147 | 0.139 | 0.033 |
| 750 | 0.187 | 0.273 | 0.073 |
| 700 | 0.244 | 0.530 | 0.189 |

**Table S3.** Summary of the electrochemical performances of SSOFC system with H_2_ fuel.

| Material | | | Fabrication | SSOFC system | | | Ref. |
| --- | --- | --- | --- | --- | --- | --- | --- |
| Electrode | | Electrolyte |  | Maximum power density  (W cm^-2^) | Polarization resistance  (Ω cm^2^) | Temp.  (^o^C) |  |
| Air side | Fuel side |  |  |  |  |  |  |
| PBMCo-^a^LSGM | | LSGM | Infiltration | 1.10 | 0.14 | 800 | This work |
| La_0.7_Ca_0.3_Cr_0.97_O_3-_*_ _*-^b^YSZ | | YSZ | Pechini | 0.05 | 0.3 | 800 | 1 |
| La_0.7_Ca_0.3_Cr_0.97_O_3-_*_ _*-^c^GDC | | LSGM | ^d^GNP | 0.21 | - | 800 | 2 |
| La_0.75_Sr_0.25_Cr_0.5_Mn_0.5_O_3-_*_ _*-YSZ | | YSZ | Solid state reaction | 0.55 | 0.18 | 950 | 3 |
| La_0.75_Sr_0.25_Cr_0.5_Mn_0.5_O_3-_*_ _*-YSZ-GDC | | YSZ | Combustion synthesis | 0.40 | 0.49 | 950 | 4 |
| Pr_0.75_Ca_0.3_Cr_0.6_Mn_0.4_O_3-_*_ _* | | YSZ | Freeze-dried method | 0.25 | - | 950 | 5 |
| Sr_2_Fe_1.5_Mo_0.5_O_6-_*_δ_* | | LSGM | GNP | 0.62 | 0.45 | 800 | 6 |
| Pr_0.5_Ba_0.4_Ca_0.1_MnO_3_  +Co-Fe (catalyst) | | LSGM+^e^LDC (buffer layer) | Pechini+Infiltration | 1.10 | - | 800 | 7 |
| ^f^NBSCF-+GDC | PrBaMn_2_O_5_ | LSGM+LDC (buffer layer) | Pechini | 0.45 | - | 800 | 8 |
| ^g^GBSCF | Ce-Pd | YSZ | Infiltration | 0.6 | 0.134 | 700 | 9 |
| GBSCF | PrBaMn_2_O_5_ | YSZ | Infiltration | 0.53 | 0.20 | 700 | 10 |
| SrCo_0.8_Fe_0.2_O_3-_*_δ_* (SCF) | Sr_2_MgMoO_6-_*_δ_* | LSGM+LDC | Combustion synthesis | 0.84 |  | 800 | 11 |
| ^a^ La_1-x_Sr_x_Ga_1-y_Mg_y_O_3-_*_δ_*, ^b^ Yittria-stabilized zirconia (YSZ), ^c^ Gadolinium doped ceria (GDC), ^d^ Glycine-nitrate process (GNP),  ^e^ Lanthanum doped ceria (LDC), ^f^ NdBa_0.5_Sr_0.5_Co_1.5_Fe_0.5_O_5+d_ (NBSCF), ^g^ GdBa_0.5_Sr_0.5_CoFeO_5+d_ (GBSCF) | | | | | | | |

**Reference**

1. B. Lin, S. Wang, X. Liu, and G. Meng, *J. Alloys Compd.*, **490**, 214–222 (2010).

2. Y. Zhang, Q. Zhou, and T. He, *J. Power Sources*, **196**, 76–83 (2011) http://dx.doi.org/10.1016/j.jpowsour.2010.07.035.

3. J. C. Ruiz-Morales, J. Canales-Vázquez, J. Peña-Martínez, D. M. López, and P. Núñez, *Electrochim. Acta*, **52**, 278–284 (2006).

4. J. C. Ruiz-Morales et al., *J. Eur. Ceram. Soc.*, **27**, 4223–4227 (2007).

5. A. El-Himri, D. Marrero-López, J. C. Ruiz-Morales, J. Peña-Martínez, and P. Núñez, *J. Power Sources*, **188**, 230–237 (2009) http://linkinghub.elsevier.com/retrieve/pii/S0378775308022027.

6. Q. Liu, X. Dong, G. Xiao, F. Zhao, and F. Chen, *Adv. Mater.*, **22**, 5478–5482 (2010).

7. S. Choi et al., *J. Mater. Chem. A*, **4**, 1747–1753 (2016) http://xlink.rsc.org/?DOI=C5TA08878J.

8. S. Sengodan et al., *Nat. Mater.*, **14**, 205–209 (2014) http://www.nature.com/doifinder/10.1038/nmat4166.

9. S. Kim et al., *ChemSusChem*, **8**, 3153–3158 (2015) http://doi.wiley.com/10.1002/cssc.201500509.

10. S. Kim et al., *Electrochim. Acta*, **225**, 399–406 (2017) http://linkinghub.elsevier.com/retrieve/pii/S0013468616327372.

11. P. Scribe et al., *Science (80-. ).*, **2**, 254–258 (2006).
